# The Role of Lymphocytic Infiltrates in the Tumor Microenvironment as a Predictive Factor for the Response to Immunotherapy in Solid Tumors: A Single-Center Experience From Romania

**DOI:** 10.7759/cureus.74194

**Published:** 2024-11-21

**Authors:** Raluca Ioana Mihaila, Adelina Silvana Gheorghe, Daniela Luminita Zob, Dana Stanculeanu

**Affiliations:** 1 Oncology Department, University of Medicine and Pharmacy, Bucharest, ROU; 2 Medical Oncology Department I, Institute of Oncology "Prof. Dr. Alexandru Trestioreanu", Bucharest, ROU; 3 Medical Oncology Department II, Institute of Oncology "Prof. Dr. Alexandru Trestioreanu", Bucharest, ROU; 4 Medical Oncology Department, Institute of Oncology "Prof. Dr. Alexandru Trestioreanu", Bucharest, ROU

**Keywords:** immunotherapy, oncology, personalized treatment, predictive biomarkers, research, solid tumors, tils, tumor-infiltrating lymphocytes, tumor microenvironment, tumor response

## Abstract

The correlation between tumor-infiltrating lymphocytes (TILs) and immunotherapy responses is an evolving field with significant clinical implications. Immunotherapy has revolutionized antineoplastic therapies, offering promising results for patients diagnosed with solid tumors. Integrating biomarkers, refining imaging techniques, and developing non-invasive methods may enhance personalized medicine, optimizing treatment strategies while minimizing adverse effects. In our study, we conducted a retrospective analysis to assess the practicality of utilizing the predictive value of tumor-infiltrating lymphocytes (TILs) in the tumor microenvironment (TME) correlating the response to immunotherapy in patients with solid tumors, comprehensively navigating through currently available data. Continued research efforts and collaboration between scientists and clinicians are essential to unlock the full potential of these biomarkers and advance the field of immunotherapy in solid tumors.

## Introduction

Immunotherapy has revolutionized antineoplastic therapies, offering promising results for patients diagnosed with solid tumors. The tumor microenvironment (TME) plays a crucial role in determining the response to immunotherapy acting as a mediator of tumor-immune system interactions. Leukocytosis and tumor-infiltrating lymphocytes (TILs) have been studied as potential biomarkers for evaluating treatment results and influencing prognosis [1-4]. This paper aims to comprehensively review the predictive value of TILs in the TME and their correlation with the response to immunotherapy in solid tumors and present the results from our clinic at the Institute of Oncology "Prof. Dr. Alexandru Trestioreanu" regarding this potential role as predictive factors. The paper will discuss the basic mechanisms, clinical implications, and future directions for the use of these biomarkers to improve treatment response and optimize personalized therapeutic strategies. Immune checkpoint inhibitors (ICIs), anti-programmed cell death 1 receptor (PD-1), anti-programmed cell death ligand (PD-L1), or anti-cytotoxic T-lymphocyte-associated antigen-4 (CTLA-4) have demonstrated significant anti-tumor activity, with tolerable safety profiles and durable responses in various neoplasia [4-6]. Currently, immunotherapy is indicated in monotherapy, in combination with immunotherapeutic agents or other antineoplastic agents (chemotherapy and targeted therapy) in the first line or subsequent lines mainly in lung cancer, melanoma, breast cancer, genitourinary cancers, or gastrointestinal cancers [1-6].

The TME is a dynamic network of cellular and non-cellular components that closely interact with tumor cells, shaping disease progression and therapy responses. It plays a critical role in tumor growth, invasion, immune evasion, and response to treatment. The cellular components of the TME include tumor cells, fibroblasts, endothelial cells, and immune cells, such as lymphocytes, macrophages, and dendritic cells. The non-cellular components include the extracellular matrix (ECM), cytokines, chemokines, and growth factors secreted by both tumor cells and stromal cells [7].

In the TME, a complex interaction between tumor cells and the immune system influences neoplastic progression or regression and consequently the response to immunotherapy. Tumor cells can create an immunosuppressive microenvironment through various mechanisms, including secretion of immunosuppressive cytokines, expression of immune checkpoint molecules, recruitment of regulatory immune cells, and alteration of antigen presentation [8-10]. By blocking these inhibitory signals, ICIs release the brakes on the immune system, improving anti-tumor immune responses. This approach has demonstrated remarkable success in various solid tumors, leading to long-lasting complete or partial remissions in certain subgroups of patients [6,8-14].

TILs play a crucial role in the antitumor immune response. TILs consist of different subsets of lymphocytes, including cytotoxic T cells, helper T cells, regulatory T cells (Tregs), and natural killer (NK) cells. It has been demonstrated that the type and functional state of TILs influence tumor behavior and response to therapy [6-10,12-14].

In addition, the extent of TILs in the TME could be determined in relation to local or systemic immunity, having prognostic value in different types of tumors. TILs have been studied as an indicator of tumor inflammation, and TIL subsets have been reported to have their own roles in cancer progression. Several studies have reported that CD8+ TILs or patients with an increased TIL infiltrate are associated with a favorable clinical prognosis. However, few data are known about the relationship between local and systemic immune responses and the correlation with response to oncological therapies and patient survival [6-14].

TILs can be classified based on their specific subtypes and spatial distribution within the TME. Effector T cells, including cytotoxic CD8+ T cells and CD4+ Th1 helper T cells, are crucial for the recognition and elimination of cancer cells. Regulatory T cells (Tregs) and CD4+ helper Th2 T cells, on the other hand, can exert immunosuppressive effects and promote tumor immune evasion. The spatial localization of TILs within the TME is also significant. Intraepithelial TILs, which are located in the tumor epithelium, have been associated with an improved prognosis in various malignant diseases. Stromal TILs, present in the tumor stroma, have also been implicated in influencing tumor behavior and response to therapy [1-4,11-14].

This paper aims to identify through this scientific research the impact of TILs and TME on the response to immunotherapy in solid tumors. The lack of such information has prompted us to share our preliminary experience in a cohort of 41 eligible cancer patients who had undergone treatment for advanced or metastatic disease. The purpose of this retrospective study was to investigate the feasibility and utility of the clinical application of determining TILs in the TME in metastatic cancer patients and identify potential benefits regarding response to ICIs. The characterization of the patient's immune parameters and the tumor microenvironment before the initiation of treatment can identify predictive biomarkers for clinical and paraclinical response and can define the subgroup of patients who do not respond to immunotherapy. It is also necessary to identify prognostic markers for patients who have an indication for immunotherapy and which can be useful for monitoring the therapeutic responses of patients.

Establishing the predictive relationship between the immune characteristics of patients, the TME and paraclinical biomarkers, and the response to immunotherapy treatments, identifying prognostic biomarkers regarding survival, quality of life, and the risk of adverse immune events, is highly important. It is the first retrospective analysis in Romania (so far) that addresses the mentioned topic.

## Materials and methods

The primary objective of this observational retrospective study is to evaluate the predictive value of tumor-infiltrating lymphocytes (TILs) in the tumor microenvironment (TME) as biomarkers for response to immune checkpoint inhibitors (ICIs) in patients with metastatic solid tumors treated at a single center in Romania. By studying the relationship between this possible biomarker and treatment outcomes, this research aims to improve the understanding of the immune landscape within the TME and its influence on the response to immunotherapy. The study aims to validate TILs independently as a predictive biomarker, analyzing them in combination with other biomarkers (e.g., PD-L1 and tumor mutational burden (TMB)) not being feasible with limited data.

The secondary objectives are to assess progression-free survival (PFS) and treatment response (complete response, partial response, and progression) in relation to TIL density, explore the feasibility and clinical utility of integrating TIL evaluation into routine practice as a predictive biomarker for immunotherapy, identify challenges in standardizing TIL assessment, and propose strategies for future validation. The ultimate goal is to identify reliable and clinically relevant predictive biomarkers that can help in the selection of patients, optimization of treatment, and development of personalized immunotherapeutic strategies. We conducted an observational retrospective, monocentric analysis, with data provided by the patients' documents treated in our Oncology Department at the Institute of Oncology. We retrospectively collected information on 41 eligible patients with histologically confirmed diagnoses of solid tumors (mostly lung cancer, renal cancer, and skin cancer) treated and followed up between 2018 and 2022. TILs were determined from the tumor tissue at the time of diagnosis and were documented in the anatomological report of the patient confirming the diagnosis. Their possible prognostic value was followed through the analysis of progression-free survival (PFS), defined as the duration (in months) from the initial administration to radiological progression based on the Response Evaluation Criteria in Solid Tumors (RECIST) version 1.1 (RECIST 1.1). The re-evaluation followed data collected from the patient file related to the clinical and biological examination and the paraclinical CT/MRI/PET-CT imaging re-evaluation according to the RECIST version 1.1 [15].

Inclusion criteria

The minimum TIL threshold for inclusion was 1% (TILs determined in the histopathological report at the time of diagnosis or in case of re-biopsy and subsequently with data related to re-evaluation available) from patients eligible for anti-PD1/PD-L1 agents for metastatic disease. Patients were selected using this specific criteria. TILs were determined in the formalin-fixed paraffin-embedded (FFPE) tissue samples by pathologist scoring of hematoxylin and eosin (H&E)-stained preparations. Patients were divided into two subgroups depending on the evaluation of TILs in TME, which will be determined from the tumor tissue: with TILs high present (>50%) and with low TIL present (<50%). The division was established to demarcate between the two groups easily [16]. The anti-PD1/PD-L1 agents used for the treatments, adjuvant or for metastatic setting, were nivolumab and pembrolizumab, in standardized dosage and schedules according to guidelines and National Health System protocols (nivolumab: 240 mg/Q2 or 480 mg/Q4, pembrolizumab: 200 mg/Q3 or 400 mg/Q6).

Exclusion criteria

Patients ineligible for immunotherapy according to data from files, patients with autoimmune diseases and chronic viral hepatitis with virus B or C, patients aged under 18 years, patients with absence of histopathological confirmation of the disease, patients with incomplete medical history or clinical data, and patients with an Eastern Cooperative Oncology Group (ECOG) performance status of 3 or 4 at the moment of the confirmation of the metastatic disease were excluded.

Considering the working hypotheses of this work, we identified possible predictive biomarkers that are easy to test, accessible, and can be used in predicting the response to immunotherapy in solid tumors and useful in monitoring patient responses. The results of this project were interpreted in correlation with the studies in the specialized literature.

After the selection of the patients and obtaining the informed consent, we collected the clinical information from the patients' medical records and pathology reports (TIL status) and evaluation of the response to the treatment (clinical, imaging, and biological), and we integrated the data through statistical tests. Informed consent of each patient was collected before inclusion. The patients were informed about the study, its goals, and the usefulness of the data that will be obtained, as well as about the confidentiality of these data. The purpose of this retrospective study was to investigate the feasibility and utility of the clinical application of using TILs as a predictive biomarker, and these data are part of the first data from the first author's PhD thesis.

Ethical aspects

The current analysis adhered to both national and international research ethics guidelines. The study complied with the ethical principles for medical research involving human subjects according to the World Medical Association Declaration of Helsinki. The clinical information had been retrieved from the patients' medical records and pathology reports. All the authors signed a data confidentiality agreement and consent to use the data for scientific purposes, and all patients signed an informed consent that enabled us to use their data for scientific purposes. Throughout the retrospective analysis, the authors adhered to ethical and medical deontology guidelines, encompassing both institutional standards and national regulations.

Statistical analysis

Statistical analyses were performed using the Statistical Package for the Social Sciences (SPSS) version 20.0 software (IBM Corp., Armonk, NY).

## Results

According to inclusion and exclusion criteria, we identified 41 eligible patients, diagnosed, treated, and followed up between 2018 and 2022. The characteristics of the enrolled patients according to the type of neoplasia, metastatic site, sex, age, and ECOG performance status are detailed in Table 1. Also, the collected data revealed that the patients were mostly over 60 years old, with good performance status (ECOG 0) (17%). Gender distribution revealed a higher share of male enrolled patients (73%) versus female patients (27%). There were 18 patients with lung cancer, eight with melanoma, five ear-nose-throat (ENT) cancers, four with non-melanoma skin cancer, and three with bladder and renal carcinoma. Most patients had visceral metastasis at diagnosis. Considering the advanced average age, the patients presented at the time of diagnosis with associated pathologies, mainly cardiac, diabetic, and pulmonary.

**Table 1:** Characteristics of the enrolled patients ENT: ear-nose-throat, ECOG: Eastern Cooperative Oncology Group

Characteristics	Subtype	All patients (N (%))
Cancer type	Lung cancer	18 (44 %)
	Melanoma	8 (20%)
	ENT cancer	5 (12%)
	Non-melanoma skin cancer	4 (10%)
	Renal cancer	3 (7%)
	Bladder cancer	3 (7%)
Metastatic situs	Visceral	26 (65%)
	Non-visceral	15 (35%)
Age	>60 years	20 (48%)
	<60 years	21 (52%)
Sex	Female	11 (27%)
	Male	30 (73%)
ECOG performance status	0	17 (42%)
	1	13 (31%)
	2	11 (27%)

**Figure 1:**
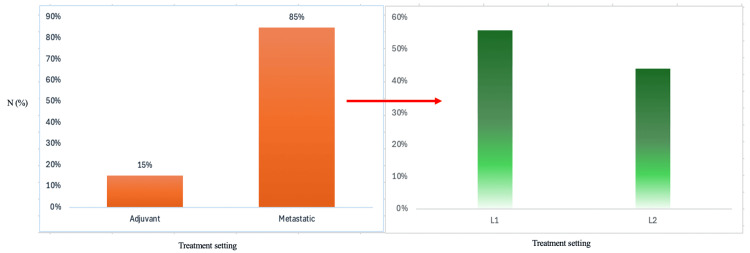
Distribution of treatment settings and lines of the patients selected in the study cohort (number (%)) L1: first line of treatment, L2: second line of treatment

Of patients identified as eligible, 56% benefited from immunotherapy as first-line (L1) treatment and 44% as second-line (L2) treatment (Figure 1). Among them, 35 patients were diagnosed with metastatic disease from the onset, and six had benefited from immunotherapy as an adjuvant therapy indication.

**Figure 2:**
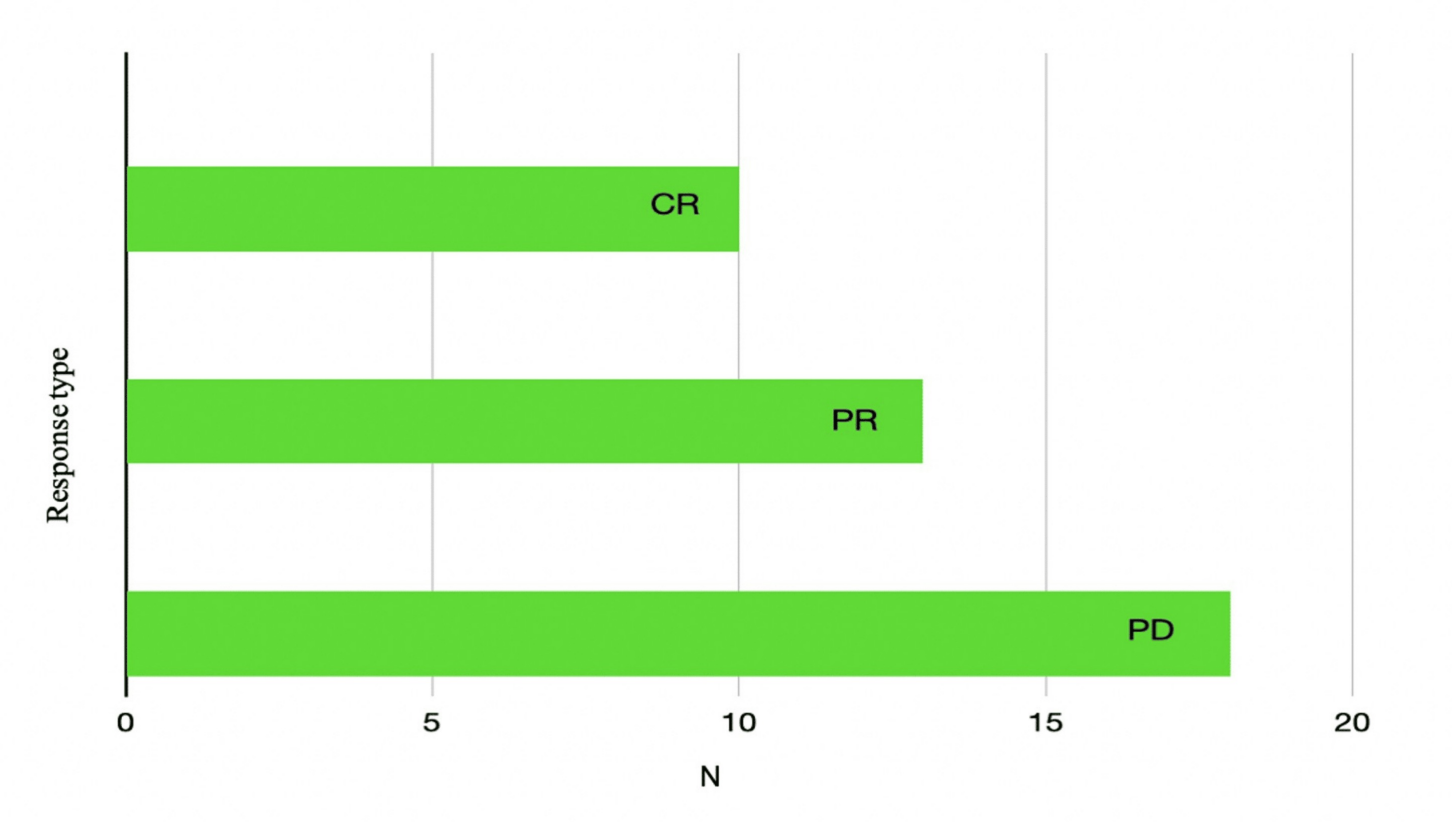
Distribution of the treatment response of the enrolled patients (number) CR: complete response, PR: partial response, PD: progressive disease

Thirteen patients had a partial response to immunotherapy, 10 patients had a complete response, and 18 patients presented progressive disease at the first re-evaluation (Figure 2). Most of the patients did not present significant immune-mediated secondary toxicities, and two patients presented severe grade IV toxicity according to Common Terminology Criteria for Adverse Events (CTCAE), one patient with severe colitis and one patient with severe hepatitis, which required definitive treatment interruption, but with the maintenance of partial or complete therapeutic response until the present moment.

**Figure 3:**
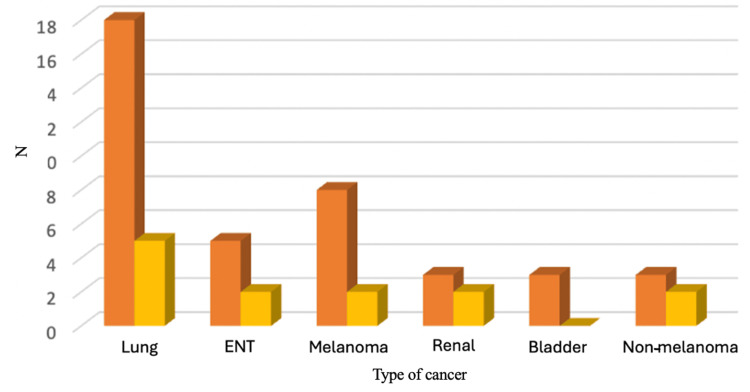
Distribution of patients at the time of diagnosis and during treatment (number) ENT: ear-nose-throat

During the follow-up of the patients, a higher proportion of survivors was identified in the case of patients with lung neoplasm or melanoma versus those with bladder neoplasm. Figure 3 shows the distribution of patients according to the primary tumor location at the time of diagnosis and after the follow-up for the mentioned period.

**Figure 4:**
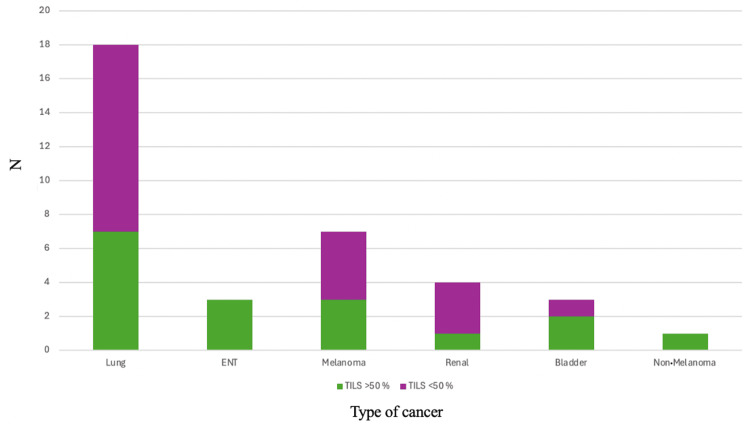
Distribution of TILs in patients according to cancer type TILs: tumor-infiltrating lymphocytes, ENT: ear-nose-throat

Of the patients who met the inclusion criteria, 36 anatomopathological results that describe the tumor microenvironment and the presence of TILs were identified; five of the 17 anatomopathological reports (out of a total of 41 eligible patients) were not very clear about the percentage value of TILs, for which they were excluded. Seventeen patients had TILs of >50%, and 19 patients had TILS of <50%. The details related to each type of cancer and the presence of TILs in eligible patients can be found in Figure 4.

**Figure 5:**
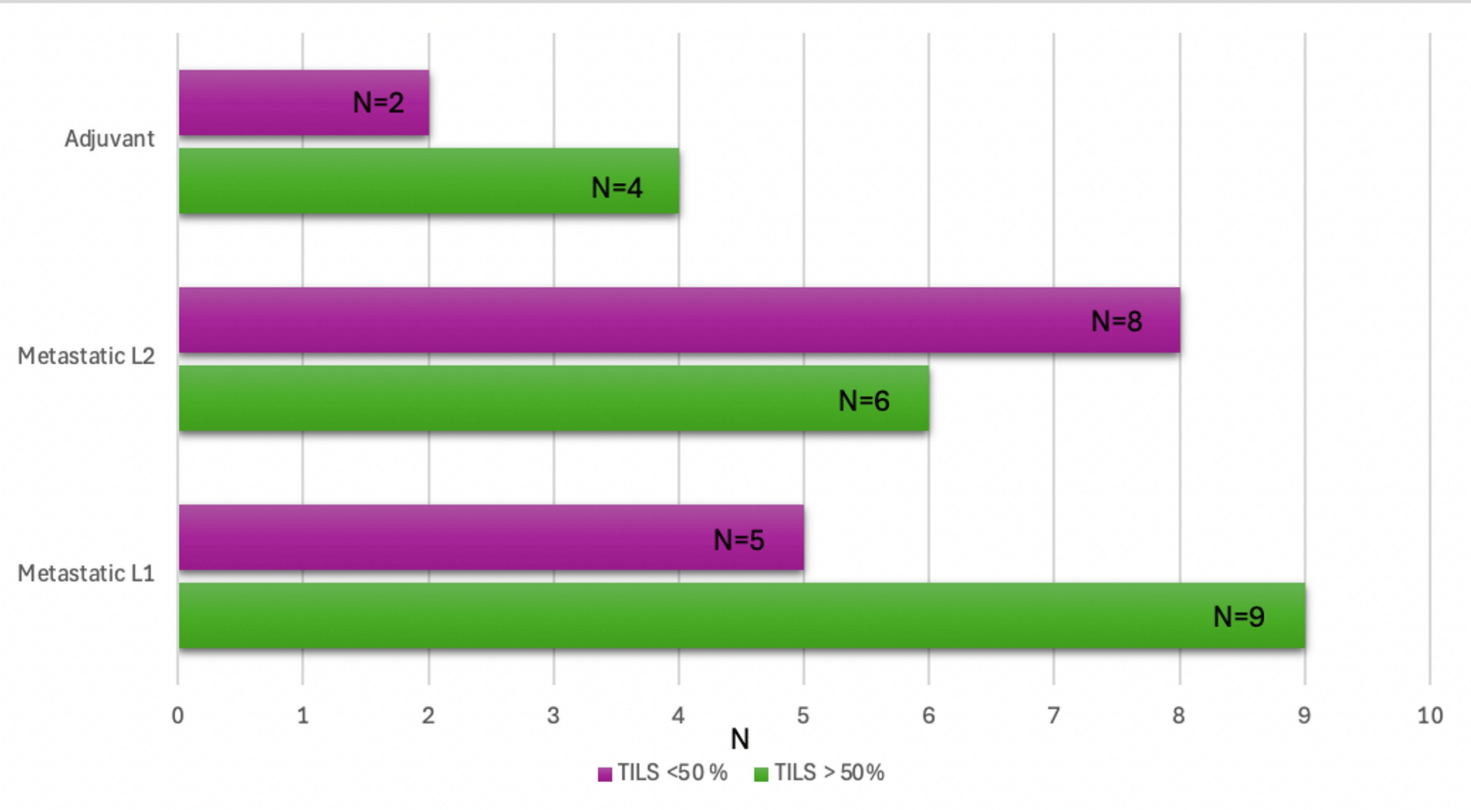
Distribution of TILs in patients according to treatment setting L1: first line, L2: second line, TILs: tumor-infiltrating lymphocytes

Of the 36 patients, most had TILs of >50% on the histopathological report in the first-line metastatic setting (nine patients), and in the second line, eight patients had TILs of <50%. In the adjuvant setting, only two patients had TILs of <50%, and four were identified with TILs above 50% (Figure 5).

**Figure 6:**
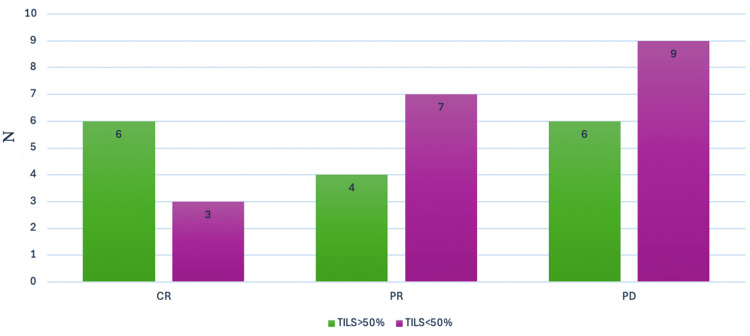
Distribution of TILs in patients according to treatment setting CR: complete response, PR: partial response, PD: progressive disease, TILs: tumor-infiltrating lymphocytes

Although the majority of patients had progressive disease after the first line of treatment, the data collected suggested that complete response was achieved in six patients and partial response in four patients with a high TIL percentage. Meanwhile, data from patients who presented progressive disease after the first line of treatment indicated that the majority had low TILs in the TME (Figure 6).

High levels of TILs, particularly cytotoxic T cells, have been linked to improved prognosis, increased overall survival, and better response to immunotherapy. According to the data published by Schalper et al., increased levels of CD3 and CD8+ TILs are associated with better outcomes in non-small cell lung cancer (NSCLC), but only CD8 is independent of other prognostic variables [17]. The presence of an active immune response within the tumor, characterized by abundant TILs, suggests an ongoing anti-tumor immune reaction and potentially favorable tumor biology [4-13]. The presence of TILs was associated with a higher probability of response to immune checkpoint inhibitors (ICIs). This observation supports the concept that pre-existing anti-tumor immune responses, as reflected by TILs, can be harnessed and potentiated by immunotherapeutic interventions [1,2,12-14].

Retrospective studies have consistently shown a positive correlation between the density of TILs and response to immunotherapy. For example, in patients with metastatic melanoma treated with ICI, high levels of TILs, particularly PD-1-positive TILs, were associated with improved response rates and longer survival.

Similar observations have been reported in other types of cancer, including NSCLC and urothelial carcinoma, highlighting the predictive value of TILs in response to immunotherapy [8-12]. Although a few patients obtained a complete response, it appears that TILs of >50% in the TME could impact this type of response. Six patients with complete response had TILs of >50% and three patients had <50%.

In NSCLC, higher levels of TILs, especially CD8+ T cells, have been correlated with better responses to immune checkpoint inhibitors [9,14,18]. In this analysis, PFS was longer in the high TIL subgroup (18.8 months (p=0.073, hazard ratio (HR): 0.90 (95% confidence interval (CI): 0.87-0.92))) compared to the low TIL subgroup (12.6 months (p=0.0856, HR: 0.83 (95% CI: 0.80-0.85))), with no statistical correlation between the presence of TILs in the TME and the progression of disease (Table 2). The presently available research data shows that TILs, combined with PD-L1 expression, improve the accuracy of predicting response to anti-PD-1/PD-L1 therapies. Donnem et al. demonstrated that high CD8+ TIL density was associated with improved outcomes in NSCLC patients treated with nivolumab or pembrolizumab [19]. Gettinger et al. published an article in 2018 that indicated the fact that combining TIL density with PD-L1 expression levels enhances the predictive accuracy of anti-PD-1/PD-L1 therapy response [20]. PDL-1 testing was not included in the analysis, and clinical data has shown that it is a potential biomarker. Further studies regarding the interaction between biomarkers in the TME should determine the possible impact of high PD-L1 TILs.

**Table 2:** Relation between TILs and PFS TILs: tumor-infiltrating lymphocytes, PFS: progression-free survival, ENT: ear-nose-throat

	Lung cancer		Melanoma		ENT		Non-melanoma skin cancer		Renal		Bladder	
	TILs > 50%	TILs < 50%	TILs > 50%	TILs < 50%	TILs > 50%	TILs < 50%	TILs > 50%	TILs < 50%	TILs > 50%	TILs < 50%	TILs > 50%	TILs < 50%
PFS (months)	18.8	12.6	20.2	9.8	9.7	5.5	10.2	4.5	20.3	14.6	8.5	7.6
P value	0.073	0.0856	0.023	0.057	0.094	0.0047	0.752	0.259	0.096	0.863	0.0678	0.450

TILs are particularly significant in melanoma, where they have been shown to predict response to immune checkpoint inhibitors such as anti-PD-1 and anti-CTLA-4 therapies. Regarding data collected from patients enrolled in this analysis, PFS was longer in the high TIL subgroup (20.2 months (p=0.023, HR: 0.72 (95% CI: 0.70-0.75))) compared to the low TIL subgroup (9.8 months), with a statistical correlation between the presence of TILs in the TME and disease progression (Table 2). Melanoma patients with a high percentage of TILs showed a better response. Other studies also indicate that high TIL density is associated with improved response rates to therapies such as pembrolizumab and nivolumab. A meta-analysis found that melanoma patients with high TIL levels had significantly better overall survival when treated with these agents. The presence of CD8+ T cells at the invasive margin of melanoma tumors is particularly predictive of response to immune checkpoint inhibitors. Tumeh et al. described in a study from 2014 that pre-existing CD8+ TILs at the invasive margin of melanoma tumors were associated with a favorable response to pembrolizumab [21]. Also, data published in this study indicated that tumor regression following therapeutic PD-1 blockade requires pre-existing CD8+ T cells that are negatively regulated by PD-1/PD-L1-mediated adaptive immune resistance. Daud et al. indicated that higher baseline TIL levels correlated with better response rates to anti-PD-1 therapy, with a significant association with progression-free survival [22]. Moreover, Erdag et al. found a strong correlation between high levels of TILs and improved survival in metastatic melanoma [23].

In head and neck squamous cell carcinoma (HNSCC), the presence of TILs is associated with better outcomes, particularly in human papillomavirus (HPV)-positive cancers. Data from the patients included emphasize that PFS was longer in the high TIL subgroup (9.7 months (p=0.094, HR: 0.78 (95% CI: 0.75-0.80))) compared to the low TIL subgroup (5.5 months), with statistical correlation between the presence of TILs in the TME and disease progression only for the low TIL subgroup (p=0.047, HR: 0.87 (95% CI: 0.85-0.90)) (Table 2). TILs have been linked to improved responses to immune checkpoint inhibitors in both HPV-positive and HPV-negative tumors. In a study published in 2016 by Mandal et al., data suggested that TILs, in conjunction with other immune markers, could predict response to checkpoint blockade therapy in both HPV-positive and HPV-negative HNSCC [24]. HPV testing results at diagnosis could not be included in the analysis as a potential co-biomarker with TILs, because they were not determined in all patient reports. Further studies regarding the correlation between biomarkers in ENT cancer patients could predict a better impact of high TILs in the TME.

Unfortunately, these first preliminary data suggest that there is no statistical correlation between the percentage of TILs and response to treatment and PFS, and thus, no positive correlation could be drawn for the other tumor types (bladder cancer, renal cancer, and non-melanoma skin cancer) (Table 2).

## Discussion

Challenges and limitations

Although the presence of TILs in the TME looks promising as a predictive biomarker for the response to immunotherapy, several challenges and limitations must be addressed. Standardization of TIL assessment methods, including scoring systems and cutoff values, is crucial for accurate and reproducible assessment. In addition, the heterogeneity of TIL subsets and their functional states requires more refined profiling techniques to better understand their specific roles in tumor immunity [6,8-11,13].

Moreover, the tumor microenvironment is dynamic and subject to spatial and temporal changes, which can influence TIL infiltration patterns. Factors such as immune checkpoint signaling, tumor heterogeneity, and immune suppression mechanisms within the TME can affect the density and functional status of TILs. Therefore, a comprehensive characterization of TME, including evaluation of other immune-related factors and molecular markers, is essential to capture the complexity of the peritumoral immune landscape and improve predictive accuracy. In addition, the interpretation of TILs as a predictive biomarker should take into account the specific tumor type and treatment context. The relationship between TILs and response to immunotherapy may vary depending on different types of tumors and therapeutic agents or other biomarkers. Therefore, it is important to validate the predictive value of TILs in certain cancer types and treatment settings to establish their clinical utility [4-6,13,14].

Until now, we have identified some expected difficulties including systematic errors (errors in the selection methods of eligible patients), errors in the instruments (TILs determined from the tumor tissue), incomplete data from the observation sheets, and no other data of potential biomarkers (PD-L1 testing and HPV testing). All these things led to the delay in collecting data in a correct and relevant way until this moment. It is also necessary to enroll a larger cohort of patients to obtain relevant data.

Integration of TILs and other biomarkers

The development of standardized protocols for TIL assessment and the integration of TIL assessment into routine clinical practice will facilitate the use of TILs as a predictive biomarker. Advances in anatomopathological and molecular techniques may further allow the precise and comprehensive characterization of TILs within the TME [6-11].

Further research is mandatory to refine the evaluation and interpretation of TILs in the context of immunotherapeutic agents. Prospective clinical studies should aim to validate the predictive value of TILs in larger cohorts of patients from different tumor types. Furthermore, studies investigating the functional characteristics of TIL subgroups, their spatial distribution, and interactions with other immune and stromal cells in the TME will provide valuable information on the underlying mechanisms of response to immunotherapy [8-10].

The integration of TILs with other biomarkers is promising for increasing the predictive value of the response to immunotherapy. Combining TIL assessment with other factors such as peripheral blood biomarkers (leukocytosis, neutrophils, lymphocytes, and monocytes), PD-L1 expression, TMB, immune gene expression profiles, and immune cell subsets may improve patient stratification and treatment selection. Multimodal biomarker approaches can provide a more comprehensive understanding of the tumor immune microenvironment and improve the accuracy of predicting response to immunotherapy [25-30].

Future directions

Standardization of biomarker evaluation methods, including cutoff values ​​and scoring systems, is essential for precise and reproducible evaluation of leukocytosis and TILs. Clear guidelines and consensus protocols must be developed to ensure coherence between different research centers and clinical settings [26-28].

Tumor heterogeneity represents a challenge in evaluating the correlation between biomarkers and response to immunotherapy and refers to the presence of distinct subclones within a tumor, each with its own molecular and immunological characteristics. TILs play a critical role in the immune system's ability to recognize and attack tumor cells [2-4]. However, low TIL density in some tumors often reflects a hostile tumor microenvironment (TME) that suppresses TIL recruitment and activity, leading to poor clinical outcomes. The mechanisms involved include a dense extracellular matrix (ECM), with tumors often having a rigid ECM composed of fibrous proteins (e.g., collagen) and glycoproteins, which impede TIL penetration into the tumor core. Additionally, abnormal vasculature, characterized by disorganized and leaky blood vessels, reduces the efficiency of TIL trafficking from circulation into the tumor tissue. Hypoxia, or low oxygen tension, can also alter TIL recruitment. Hypoxia-induced factors promote the exclusion of immune cells from the tumor bed. The density of TILs can vary in different regions of the tumor, influencing the general immune landscape of the tumor [2-4,7].

Testing of the clinical validity of biomarkers requires determining the extent to which the biomarker predicts the clinical outcome and response to a specific treatment. Standardization of TIL measurement addresses the lack of protocols for assessing TILs. Current research, such as guidelines proposed by the International Immuno-Oncology Biomarker Working Group, could offer precision in determining the best option over the assessment of TILs. Semi-quantitative H&E-based scores may suffer from low precision and poor inter-observer reproducibility if no clear guidance exists, while digital quantification of immunohistochemistry (IHC)-stained sections may produce different results due to the inaccurate measurement of the test variable without controlled calibration [31,32].

Variations in immune infiltrates in different regions of the tumor can influence the global predictive value of leukocytosis and TILs. Integrated biomarker approaches that consider multiple factors, such as leukocytosis, TILs, PD-L1 expression, TMB, and immune gene expression profiles, have the potential to improve patient outcomes by tailoring treatments to individual characteristics [25-30,33-36]. Integration of TILs combined with other biomarkers such as PD-L1 and TMB often improves predictive accuracy. According to data published in 2017, Zhou et al. identified that circulating soluble programmed death ligand-1 (sPD-L1) was a prognostic biomarker that may predict outcomes for subgroups of patients receiving checkpoint inhibitors [33]. A meta-analysis by Goodman et al. on TMB as a predictor supports this integration, with higher TMB predicting favorable outcomes to PD-1/PD-L1 blockade across diverse tumors [34].

In the future, a liquid biopsy, which is a non-invasive diagnostic procedure that involves the isolation of circulating biomarkers such as circulating tumor cells, cell-free DNA (cfDNA), and microRNA (miRNA), would be very useful considering recent advances in genomic sequencing (next-generation sequencing (NGS)) and polymerase chain reaction (PCR)-type analysis, to provide basic information about tumors and monitor the response to therapies [36]. According to Lee et al., liquid biopsies could represent the future standard testing method (e.g., circulating tumor DNA) in monitoring immunotherapy responses [35]. However, everything involves very high costs, which is not applicable in real life. The measurement of the usual inflammatory biomarkers is simple and convenient regarding the long-term costs [34-37].

Incorporating TILs as a predictive biomarker in clinical practice can have significant implications for patient outcomes. By accurately identifying patients who are more likely to benefit from immunotherapy, unnecessary treatments can be avoided, and patients can be directed to alternative therapeutic strategies. In addition, identifying patients at higher risk of treatment resistance or immune-related adverse events may allow for proactive management and personalized interventions [6-8,35-37]. Future studies should consider spatial heterogeneity and develop methods to capture the immune landscape comprehensively.

## Conclusions

Tumors have unique immunological characteristics, and response to immunotherapy can vary. The mechanisms underlying the response to immunotherapy may differ, underlining the importance of studying these correlations in specific tumor contexts. In addition, treatment modalities, such as the type of immunotherapy agents used, the combination with other therapies, and the treatment schedule, may influence the relationship between TILs and response to immunotherapy. The modulation of immune cell infiltration by immunotherapy must be taken into account when evaluating treatment results. The data from the present analysis highlighted that a single biomarker is not enough to identify patients' response to immunotherapy and that TILs have a high potential predictive characteristic. Therefore, correlating them could be useful, but it has a complex and multifaceted relationship. Future research efforts should focus on validation studies, mechanistic investigations, integrated biomarker approaches, dynamic assessment, and personalized medicine to fully exploit the potential of these biomarkers in guiding immunotherapy treatment decisions. By integrating several biomarkers, studies can improve the accuracy of patient selection and treatment decision-making. These advances have the potential to revolutionize the field of immunotherapy and improve patient outcomes by optimizing treatment strategies, reducing unnecessary treatments, and proactively managing treatment-related toxicities.
